# An Unusual Case of Thromboangiitis Obliterans With Concurrent Deep Vein Thrombosis and Pulmonary Embolism

**DOI:** 10.7759/cureus.96506

**Published:** 2025-11-10

**Authors:** Zahra Vaezi, Donica L Baker

**Affiliations:** 1 Internal Medicine, St. Luke's Hospital, Chesterfield, USA; 2 Rheumatology, St. Luke's Hospital, Chesterfield, USA

**Keywords:** autoamputation, buerger’s disease, deep vein thrombosis (dvt), pulmonary embolism (pe), thromboangiitis obliterans

## Abstract

Buerger’s disease (thromboangiitis obliterans (TAO)) is a rare, smoking-related vasculitis that primarily affects distal extremity vessels but can occasionally involve the venous system. We report a 45-year-old heavy smoker with chronic obstructive pulmonary disease (COPD), Raynaud phenomenon, prior digital autoamputation, and necrotic fingertip lesions who presented with dyspnea, hemoptysis, rib pain, and thigh discomfort. He was tachycardic, tachypneic, and hypoxic, with digital gangrene and synovitis. Laboratory studies showed leukocytosis, elevated inflammatory markers, and positive antinuclear antibody (ANA). Duplex ultrasound revealed extensive bilateral deep vein thrombosis (DVT), and CT angiography confirmed acute pulmonary embolism (PE) with right heart strain.

The patient was diagnosed with TAO complicated by systemic thromboembolic disease and treated with anticoagulation alongside strict smoking cessation counseling. This case underscores that although TAO is classically a peripheral arterial disease, venous involvement can predispose patients to DVT and PE, especially in the setting of systemic inflammation. Clinicians should maintain suspicion for PE in TAO patients with unexplained respiratory symptoms and emphasize tobacco cessation as essential for preventing recurrence and progression.

## Introduction

Buerger’s disease, also known as thromboangiitis obliterans (TAO), is a rare, segmental, inflammatory, and thrombotic vasculitis that primarily affects small- and medium-sized arteries and veins, typically in the distal extremities. First described by Leo Buerger in 1908, the disease is most commonly seen in young adult males with a strong history of tobacco use [1]. However, more recent case series and reports suggest that the clinical spectrum may be broader, occasionally affecting women and patients with comorbidities such as diabetes mellitus [2,3]. The pathogenesis of TAO is not entirely understood but appears to involve an interplay between genetic susceptibility, tobacco exposure, immune-mediated endothelial injury, and coagulation abnormalities. Histologically, it is characterized by a highly cellular thrombus with relative sparing of the internal elastic lamina and a panvasculitis picture involving both arteries and veins [2].

Several hypotheses have been proposed to explain the underlying mechanisms driving TAO. Serotonin dysregulation has been implicated in promoting vasospasm and thrombus formation in TAO patients [4]. Furthermore, autoimmune processes appear to play a significant role, particularly in individuals with specific human leukocyte antigen (HLA) phenotypes, where tobacco antigens may act as immunologic triggers [5]. Neurovascular dysfunction, as explored in treatments involving spinal cord stimulation, also supports the notion that vasomotor instability may contribute to ischemic episodes in TAO [5]. The disease classically affects the lower limbs but can occasionally involve visceral arteries, with rare but severe presentations such as mesenteric ischemia and small bowel infarction [6]. A recent comprehensive review of 91 patients from Iran further illustrated the diverse presentation of TAO, including both arterial and venous thromboses [7].

Although TAO is primarily regarded as a peripheral vascular disorder, emerging literature hints at possible systemic vascular involvement, raising the question of whether central thromboembolic complications, such as pulmonary embolism (PE), might occur in some patients. PE typically arises from thrombus formation in the deep veins of the legs or pelvis, dislodging and migrating to the pulmonary arteries. The involvement of superficial and deep veins in TAO, while less emphasized in historical descriptions, has been documented in several studies and case reports. In this setting, a hypercoagulable state, either intrinsic to the disease or exacerbated by tobacco-induced endothelial injury, could contribute to venous thrombus propagation and embolization. While direct associations between TAO and PE are rare and largely undocumented in large cohorts, the presence of systemic ischemic phenomena such as coronary artery disease [8] and bowel infarction may represent a broader disease process than previously assumed.

Given the morbidity and mortality associated with PE, it is important to consider the possibility of this complication in select TAO patients, particularly those with extensive venous involvement or unexplained cardiopulmonary symptoms. Although no large-scale studies have directly evaluated the incidence of PE in TAO, isolated reports and the systemic nature of some TAO presentations underscore the need for heightened clinical suspicion. Further research is needed to establish the prevalence of venous thromboembolism, including PE, in this population and to determine whether prophylactic or diagnostic strategies should be considered in patients with advanced or atypical TAO phenotypes. Understanding these connections may ultimately improve patient outcomes through more comprehensive risk stratification and targeted management [1].

## Case presentation

The patient is a 45-year-old Caucasian male with a complex medical history, including tobacco use (one pack per year), chronic obstructive pulmonary disease (COPD) (Global Initiative for Chronic Obstructive Lung Disease (GOLD) stage II), recurrent pneumonia, unprovoked deep vein thrombosis (DVT), Raynaud phenomenon complicated with necrotic autoamputation of the fingertip, and hypertension, who presented with two weeks of progressive shortness of breath, dyspnea on exertion, intermittent hemoptysis, and left-sided rib pain. He reported chills and severe bilateral thigh pain.

**Figure 1:**
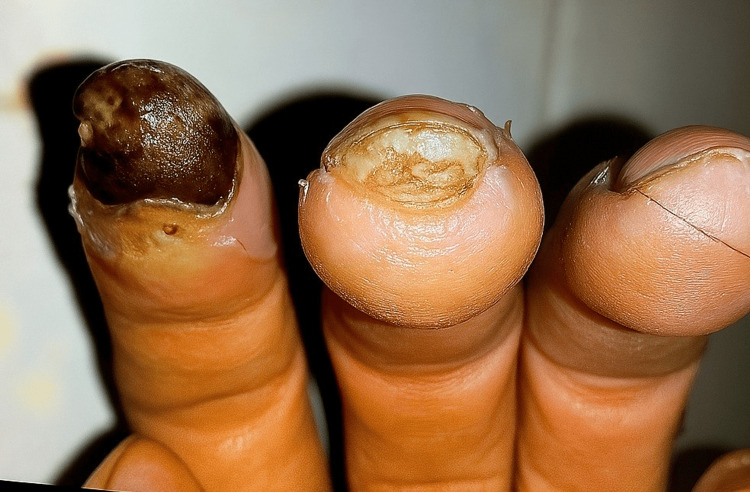
Autoamputation and necrosis of the second fingertip

He denied alcohol use and had a remote history of marijuana and stimulant use, but no IV drug use. The review of systems was notable for fatigue, night sweats, finger discoloration with exposure to cold, chronic cough, joint swelling, and digital necrosis. On admission, vital signs were significant for a heart rate of 114 beats per minute, a respiratory rate of 35 per minute, and an oxygen saturation of 50%. The physical exam showed diminished bilateral breath sounds, regular cardiac rhythm, no skin tightening, trace swelling, and warmth of the bilateral metacarpophalangeal (MCP) and proximal interphalangeal (PIP) joints, and autoamputation and gangrene of the left second fingertip (Figure 1).

Laboratory evaluation showed leukocytosis (27.3 K/µL; 16% bands, 72% neutrophils), elevated erythrocyte sedimentation rate (32 mm/h), and C-reactive protein (1.9 mg/dL) (Table 1). 

**Table 1:** Hematologic lab test results MCV: mean corpuscular volume; ESR: erythrocyte sedimentation rate; CRP: C-reactive protein

Parameters	Result	Reference Range	Unit
WBC	27.3	4.3-10.0	K/uL
Lymphocyte	84%	15.0-45.0	%
Neutrophil	5%	40.0-80.0	%
Monocyte	10.0%	0.0-12.0	%
Hemoglobin	15.6	13.6-15.6	g/dL
Hematocrit	50.3	40-48	%
MCV	86.9	79-93	fL
Platelet	301	165-415	1000/mL
ESR	32	0-15	mm/h
CRP	1.9	0-0.9	mg/dL

Infectious studies, including multiplex respiratory polymerase chain reaction (PCR), urinalysis, and cultures, were negative. Biochemistry revealed normal renal and liver function with elevated N-terminal pro-B-type natriuretic peptide (NT-proBNP) (3,260 pg/mL) (Table 2). Autoimmune testing showed antinuclear antibody (ANA) 1:1280 with negative anti-dsDNA, extractable nuclear antigen (ENA) (Sjӧgren’s syndrome-related antigen A (SSA), Sjӧgren’s syndrome-related antigen B (SSB), ribonucleoprotein (RNP), Smith (Sm)), antineutrophil cytoplasmic antibody (ANCA), and rheumatoid factor.

**Table 2:** Biochemistry lab test results anti-CCP: anti-cyclic citrullinated peptide; BUN: blood urea nitrogen; NT-proBNP: N-terminal pro-B-type natriuretic peptide

Parameters	Result	Reference Range	Unit
ANA	1: 1280	< 1:40	
Anti-CCP Ab	6	<20	U/mL
Troponin-I	0.03	0- 0.014	ng/mL
NT-proBNP	3,260	<125	pg/mL
Sodium	135	135-145	mEq/L
Potassium	4.2	3.5-5.0	mEq/L
Chloride	94	96-106	mEq/L
BUN	18	7-20	mg/dL
Creatinine	0.8	0.7-1.3	mg/dL
Calcium	8.8	8.5-10.5	mg/dL

**Figure 2:**
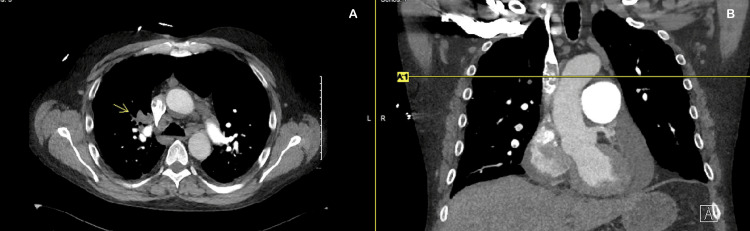
Right upper lobe pulmonary embolism, right pulmonary artery occlusion (yellow arrow) A: CT pulmonary angiogram, bilateral pulmonary emboli; B: venogram, extensive femoropopliteal DVT. DVT: deep vein thrombosis

**Figure 3:**
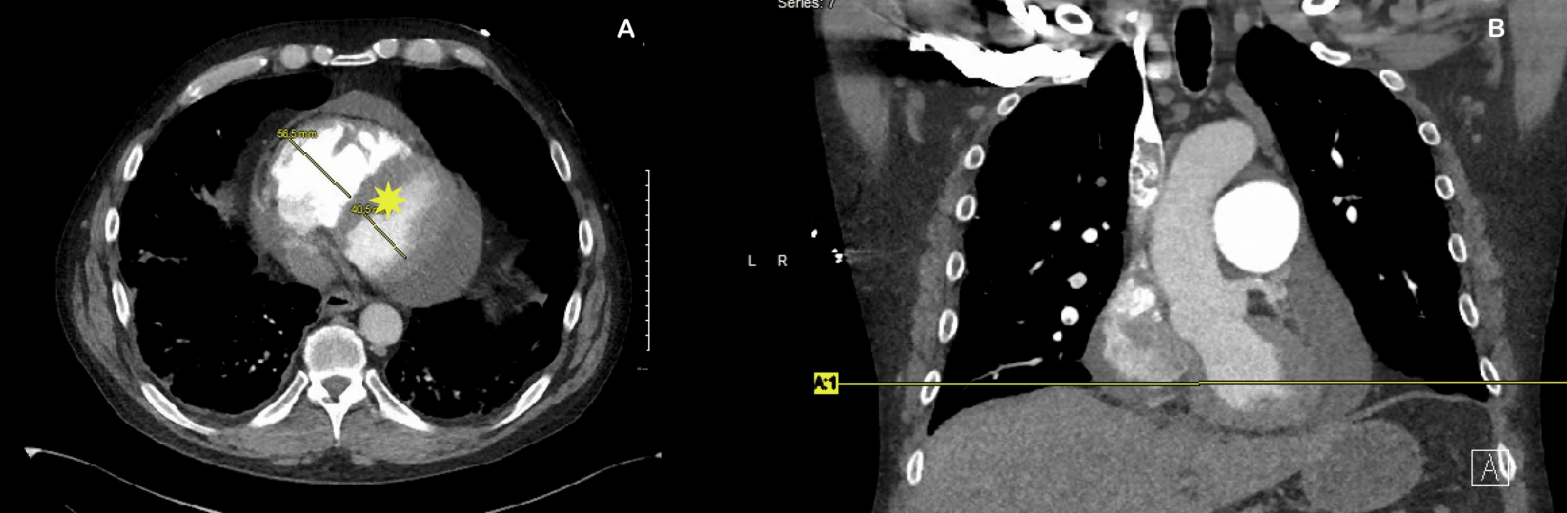
Right heart strain, D sign on axial chest view (yellow asterisk) A: CTA, segmental tibial occlusions consistent with TAO; B: histopathology, preserved internal elastic lamina with inflammation (H&E ×100). TAO: thromboangiitis obliterans

Bilateral venous duplex ultrasonography revealed acute DVT involving the right femoral, popliteal, gastrocnemius, posterior tibial, and peroneal veins. CT angiography of the chest demonstrated acute PE affecting the right upper, middle, and lower lobes with right-heart strain and pulmonary infarcts (Figures 2, 3). Thrombophilia screening (protein C, protein S, antithrombin III, and antiphospholipid antibodies) was unremarkable. Malignancy was excluded with CT of the chest, abdomen, and pelvis and age-appropriate cancer screening. Diabetes mellitus was ruled out with normal HbA1c and fasting glucose levels.

The patient was treated with therapeutic enoxaparin followed by warfarin for long-term anticoagulation and counseled on immediate and complete smoking cessation. He was offered nicotine replacement therapy (patch) and referral to a smoking-cessation program.

## Discussion

This case illustrates an uncommon but clinically significant manifestation of TAO complicated by diffuse DVT and acute PE. Although TAO is typically a peripheral vasculitis confined to the extremities, venous thrombosis occurs in a subset of patients [7]. The combination of extensive DVT and PE, in the absence of conventional risk factors such as immobility, surgery, or malignancy, suggests that TAO may, in certain cases, involve systemic vascular inflammation. This case highlights an uncommon but clinically significant manifestation of Buerger’s disease (TAO) presenting with acute PE, DVT, and ischemic complications. TAO is classically a non-atherosclerotic, inflammatory vasculopathy involving the small- and medium-sized arteries and veins of the extremities, most frequently seen in young male smokers [7]. Our patient, despite being slightly older than the typical age range, had multiple risk factors, including heavy tobacco use, Raynaud phenomenon, and digital necrosis, all of which support the diagnosis of TAO. His disease course was further complicated by bilateral DVT and acute PE, a constellation rarely reported in the context of Buerger’s disease, suggesting a more systemic vascular involvement than traditionally recognized.

Although the pathophysiology of TAO is not fully elucidated, several mechanisms may explain its potential to cause thromboembolic events. TAO involves both arteries and veins, and venous thrombosis is not uncommon in these patients [7]. Histologically, the disease is characterized by segmental thrombosis and inflammation with relative preservation of the internal elastic lamina, implicating immune-mediated injury possibly triggered by tobacco antigens [6]. The presence of systemic inflammation and leukocytosis, as seen in our patient, may further enhance thrombotic risk through endothelial injury and hypercoagulability. Serotonin dysregulation has also been proposed as a contributor to vasospasm and thrombogenesis, adding to the multifactorial risk of thrombosis in TAO [9].

While arterial occlusion and limb ischemia are well-known sequelae of TAO, PE remains a rare and underrecognized complication. There are no large cohort studies linking TAO to PE, but systemic ischemic involvement, including coronary artery disease [8] and mesenteric infarction [9,10], has been reported. These findings suggest that Buerger’s disease may, in select patients, extend beyond the extremities and involve the central vasculature. In our case, the presence of extensive femoral and popliteal DVT, followed by segmental pulmonary emboli and right heart strain, supports the possibility of a broader disease phenotype. Notably, this occurred in the absence of traditional risk factors for PE, such as immobility, recent surgery, or malignancy.

Management of TAO relies primarily on the complete and immediate cessation of tobacco use, which is both therapeutic and preventive. Continued smoking is strongly associated with progression to limb loss and worsening thrombotic complications [2,7]. In addition to smoking cessation, anticoagulation is essential in cases of venous thromboembolism. Our patient was started on therapeutic enoxaparin followed by warfarin, following current guidelines for PE with right heart strain. The co-occurrence of Raynaud phenomenon and elevated ANA also raises the possibility of overlapping autoimmune vasculopathy, although his clinical picture remains most consistent with TAO. In rare and refractory cases, interventions such as spinal cord stimulation have been explored to manage ischemic pain [6], although these were not indicated in this patient.

This case underscores the importance of considering systemic thromboembolic disease in patients with advanced Buerger’s disease, particularly when there is venous involvement or cardiopulmonary symptoms. While PE is not a well-established complication of TAO, this case adds to a growing body of literature suggesting that such presentations, though rare, may occur. Early recognition and intervention, including anticoagulation, tobacco cessation, and appropriate vascular imaging, are crucial to improving outcomes in this complex patient population.

## Conclusions

This case illustrates a rare but clinically significant presentation of Buerger’s disease complicated by extensive DVT and acute PE. While TAO is typically considered a peripheral vascular disease limited to the extremities, this case supports the growing recognition that it may, in select individuals, have systemic thromboembolic manifestations. The coexistence of arterial and venous involvement, along with pulmonary vascular complications, underscores the importance of maintaining a high index of suspicion for PE in TAO patients presenting with respiratory symptoms and risk factors for venous thromboembolism.

Prompt diagnosis and management, including therapeutic anticoagulation and strict tobacco cessation, are essential to prevent further morbidity. This case further emphasizes the need for clinicians to consider TAO in the differential diagnosis of young smokers with ischemic limb changes, particularly when accompanied by thromboembolic events beyond the extremities. Additional studies are warranted to better define the incidence, pathophysiology, and optimal management strategies for systemic involvement in Buerger’s disease.
